# The Toxic Effects of Environmental Domoic Acid Exposure on Humans and Marine Wildlife

**DOI:** 10.3390/md23020061

**Published:** 2025-01-29

**Authors:** Ami E. Krasner, Margaret E. Martinez, Cara L. Field, Spencer E. Fire

**Affiliations:** 1 Florida Institute of Technology, Department of Ocean Engineering and Marine Sciences, Melbourne, FL 32901, USA; sfire@fit.edu; 2 The Marine Mammal Center, Sausalito, CA 94965, USA

**Keywords:** domoic acid toxicosis, harmful algal blooms, biotoxin, marine wildlife, amnesic shellfish poisoning, glutamate receptor, sentinels

## Abstract

Biotoxins produced by harmful algal blooms (HABs) are a substantial global threat to ocean and human health. Domoic acid (DA) is one such biotoxin whose negative impacts are forecasted to increase with climate change and coastal development. This manuscript serves as a review of DA toxicosis after environmental exposure in humans and wildlife, including an introduction to HAB toxins, the history of DA toxicosis, DA production, toxicokinetic properties of DA, susceptibility, clinical signs, DA detection methods and other diagnostic tests, time course of toxicosis, treatment, prognostics, and recommendations for future research. Additionally, we highlight the utility of California sea lions (CSLs; *Zalophus californianus*) as a model and sentinel of environmental DA exposure.

## 1. Introduction to Harmful Algal Biotoxins

Biotoxins naturally produced by harmful algal blooms (HABs) are increasingly documented worldwide as agents of disease impacting marine wildlife and public health [1]. The apparent escalation in HAB events over the last several decades may be due to improved awareness, monitoring efforts, and detection methods [1,2,3]. Climatic shifts, aquatic eutrophication, overfishing, marine regime shifts, and industrialization are suspected contributors to an expanding distribution, incidence, intensity, and/or number of toxicogenic HAB species in certain regions [3,4,5]. Harmful algal biotoxins are a primary health threat to marine wildlife in the USA and a major cause of strandings and mass mortality events [6,7].

There are at least a dozen HAB toxins of major, minor, and/or emerging concern to human and animal health worldwide [2]. Simultaneous HAB toxin exposure may occur in humans and marine wildlife [8,9,10,11,12], as well as in the seafood they consume [13,14]. Exposure to multiple HAB toxins may increase individual potencies [15]. Seafood can also be contaminated with concurrent harmful agents (e.g., heavy metals, *Vibro* spp.) [16].

Routes of exposure to HAB toxins are primarily via oral ingestion of contaminated seawater or prey but can include dermal contact or respiratory inhalation of aerosolized toxins [2]. Prey can bioaccumulate HAB toxins prior to vectoring these potentially harmful agents to higher trophic-level predators [2]. Harmful algal bloom toxins typically disrupt cellular signaling pathways through binding to membrane (i.e., receptor or channel) or intracellular proteins, commonly resulting in neurotoxicity, cardiotoxicity, hepatotoxicity, gastrointestinal (GI) upset, or tumor promotion [2]. Observable health effects can be immediate (acute toxicosis) or delayed weeks to years after exposure or accumulate over multiple exposures (chronic toxicosis) [17,18,19]. Hydrophilic toxins are rapidly depurated within hours to days, while hydrophobic toxins may persist for weeks to months [2]. The GI tract and kidneys often act as primary routes of excretion. Biotoxin kinetics and effects are impacted by (1) exposure route, duration, dose, frequency, and history, (2) biotoxin properties (e.g., hydrophilicity and potency), (3) anatomy and physiology of the exposed taxa, and (4) underlying health status, developmental stage, and sex of the exposed individual. Data is lacking regarding toxicokinetic properties, HAB toxin interactions, subtle health impacts or those from repeated exposure(s), and diagnostic confirmation of previous exposure(s) in marine wildlife and humans [19].

This manuscript focuses on the biotoxin domoic acid (DA) and related disease in humans and marine wildlife. Domoic acid can cause acute and chronic neurobehavioral deficits, cardiac disease, reproductive failure, and mortality in numerous species [2,17,18,19]. Domoic acid exerts its harmful effects by mimicking the excitatory neurotransmitter L-glutamate in neural and extra-neural tissues [2,17]. Research has focused on characterizing disease in the central nervous system (CNS) of laboratory models of DA toxicosis, as well as environmentally exposed marine wildlife and humans with frequent DA consumption. The history of DA toxicosis, DA production, toxicokinetics, susceptibility, clinical signs, diagnostic tests, medical treatment options, prognosis, and future recommendations in DA-related clinical research are discussed. Additionally, we highlight the California sea lion (CSL; *Zalophus californianus*) as a model and sentinel of environmental DA exposure.

## 2. History of DA Toxicosis

Domoic acid was first isolated from a red macroalgae in Japan in the 1950s [20]. For several decades, DA was used to treat GI parasites in humans. Minimal to no side effects were reported, except for mild headaches, GI upset, and fatigue with doses over 0.66 mg/kg body weight [21,22,23]. The toxin was recognized as a public health threat in 1987 after causing acute poisoning in over 100 individuals, leading to nineteen hospitalizations and four deaths [24,25]. The outbreak of human illness, called Amnesic Shellfish Poisoning (ASP) from the distinctive anterograde memory loss described in 25% of patients, occurred after consumption of contaminated mussels in Prince Edward Island, Canada [25]. Amnesic shellfish poisoning was defined as the development of GI signs within 24 h and/or neurologic signs (e.g., amnesia, headache, confusion, hallucinations, seizures, or coma) within 48 h after DA exposure [24]. Aged individuals and those with underlying health conditions (i.e., diabetes mellitus, hypertension, or chronic kidney disease) were most severely affected [26]. Except for an unconfirmed outbreak in Washington State in 1991 [26], ASP has been minimized through monitoring DA levels in seafood for public consumption [19]. Recreational and commercial seafood closures occur in the USA, Canada, Australia, New Zealand, and the European Union if levels exceed the regulatory limit (20 ppm shellfish tissue; 30 ppm Dungeness crab viscera) [27,28,29]. Yet, this limit is not specific to age, sex, body weight, DA consumption rate and exposure history, pregnancy, lactation, or underlying health status; thus, it may inadequately protect sensitive or high-risk groups or those with long-term, low-level ingestion [19].

A chronic condition secondary to DA toxicosis was first identified when an elderly patient who recovered from severe ASP presented one year later with temporal lobe epilepsy (TLE), presumed to be a delayed ASP side effect [30]. Native American tribe members, participants of the Communities Advancing the Studies of Tribal Nations Across the Lifespan (CoASTAL) cohort [31], exhibited memory deficits from repeated DA exposure below the safety threshold [19]. Thus, tolerable daily intake (TDI) recommendations were modified to 0.003 mg/kg/day for adults or a maximum of 15 razor clams/month, particularly for young, aged, pregnant, or renal-compromised individuals [29,32].

In numerous marine wildlife species, DA has been associated with acute and chronic illness, death, mass stranding events, and population declines, including those that are a conservation priority. In 1991, around 150 seabirds in Monterey Bay, California, developed acute GI and neurologic illness or died from the toxin [33,34]. Subsequently, DA exposure or toxicosis has been identified in numerous seabird species spanning the Pacific coast from Alaska to Mexico, as well as Rhode Island and Spain [35,36,37,38,39]. Since at least 1998, CSLs have stranded from acute DA toxicosis (an immediate consequence of high-level exposure) or domoic acid-induced epileptic disease (a condition caused by repeated/chronic DA exposure or a delayed consequence of previous sublethal acute exposure), and they are the most well-documented, systemically researched, and visibly impacted marine species [28,40]. Primarily seasonal, nearly annual DA-producing blooms impact dozens to hundreds of CSLs at a time in California, with more isolated cases of DA toxicosis in Pacific Northwest populations [36]. The toxin has caused similar disease outcomes in the threatened (federally listed) keystone subspecies southern sea otters (SSOs; *Enhydra lutris nereis*) in central California [41,42]. Other marine wildlife experience less frequent, subtle, or unclear impacts from DA exposure. The first known cases of DA toxicosis in cetaceans involved several species that were stranded with marked neurologic signs during a 2002 marine mammal unusual mortality event (UME) in southern California [43]. Since then, DA exposure has been documented in other cetaceans worldwide, correlated with mass stranding events and an increased vulnerability to injury/mortality from fisheries interaction and vessel strikes, and linked to the failed recovery of several species of conservation concern, including northern (*Eubalaena glacialis*) and southern (*Eubalaena australis*) right whales [9,44,45,46,47,48,49,50,51]. The toxin was also suspected to have caused the strandings and subsequent deaths of two endangered marine reptiles: green sea turtles (*Chelonia mydas)* in Florida and leatherback sea turtles (*Dermochelys coriacea*) in California [52,53,54].

## 3. Domoic Acid Production

**Figure 1:**
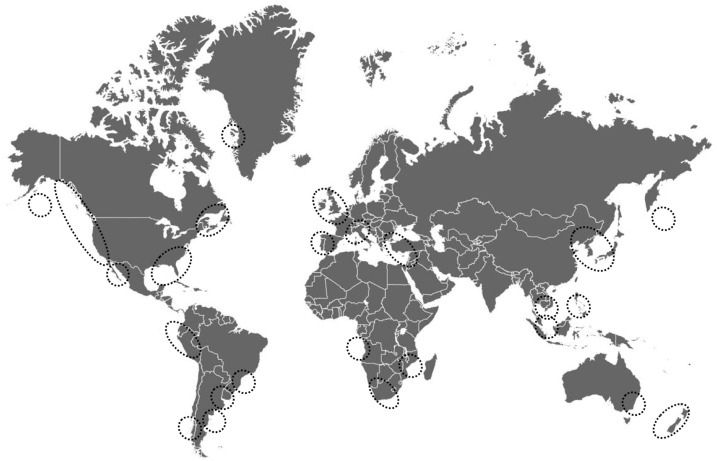
Worldwide distribution of domoic acid-producing blooms (dashed circles) (Adapted from [2]).

Domoic acid is naturally synthesized by at least half a dozen species of red macroalgae and over twenty-five species of pennate diatoms in the genus *Pseudo-nitzschia* and related *Nitzschia* species [1,55]. The toxin is produced in diverse marine waters worldwide, including the coast and open ocean, as well as arctic and equatorial environments. Domoic acid has been detected in the offshore waters of all continents but Antarctica [4,9,10,13,45,56,57,58,59,60,61,62,63,64,65,66,67,68,69] (Figure 1). Domoic acid production may be occurring with increasing frequency, potency, duration, and geographic extent, as well as by a greater number of algal species in some regions of North America [1,3]. Detecting the toxin can be challenging in offshore environments and regions with bloom patchiness. Monitoring DA production can also be challenging in communities or nations with limited access to the necessary resources.

Triggers of bloom formation and DA synthesis are multifactorial and complex, as there are regional, seasonal, anthropogenic, environmental, and hydrographic influences [70,71,72]. While DA is detectable year-round in many regions, seasonal blooms occur in California (late spring to fall), the northeast USA (late fall to winter), and the Gulf of Mexico (spring) [1,66,73,74,75]. Triggers may be linked with climatic cycles or extreme ocean events, such as the “Warm Water Anomaly“ in 2015–2016 along the USA Pacific coast, which was associated with the most toxic, extensive, and persistent DA-producing bloom ever recorded [36]. As ocean warming, acidification, and nutrient levels are major contributors to HAB blooms and toxin synthesis, the global threat of exposure is expected to magnify with climate change projections and urban expansion.

Domoic acid can persist in pelagic and benthic water and food webs [44,76], including marine snow and sediments [77,78], representing exposure sources in lieu of toxigenic blooms. The toxin is stable under ambient environmental conditions but may degrade upon exposure to ultraviolet radiation, oxygen, or extreme pH or temperature fluctuations [79]. Conventional cooking of DA-contaminated seafood only minimally degrades the toxin [80].

## 4. Toxicokinetic Properties of DA

**Figure 2:**
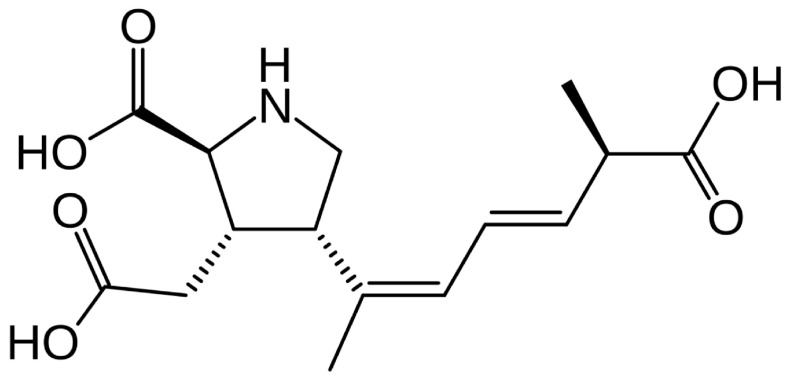
The structure of DA, an excitatory amino acid and harmful algal bloom (HAB) toxin.

Domoic acid is a polar, hydrophilic, excitatory amino acid (EAA) that is similar in biochemical structure and function to kainic acid (KA), L-aspartate, and L-glutamate [1,81] **(Figure 2**). Kainic acid is a biotoxin naturally produced by a macroalgae [82]. L-aspartate and L-glutamate are excitatory neurotransmitters in vertebrates, but L-glutamate is dominant. Both DA and KA can bind to glutamate receptors (GluRs) and mimic glutamate’s biological activity [83,84]. However, DA is three times more potent than KA and up to 100× more potent than L-glutamate [85]. Though there are about a dozen known DA isomers with variable potency, isomers are not considered a substantial threat to humans and marine wildlife due to their lower GluR affinity and environmental concentrations [19,25].

Toxicokinetic data is primarily from healthy laboratory animals (murine, finfish, and nonhuman primates) administered parental DA. Extrapolation to environmental exposure is complicated by differential sensitivity based on species, exposure route and amount, exposure duration, and individual health history. Rodents and finfish, two of the most common models for DA toxicosis, are more resistant to the toxic effects of DA than humans or marine wildlife [86,87]. There is variable uptake, storage, and elimination between taxa [88]. Laboratory studies utilize pure DA, but seafood may also contain isomers or other contaminants with varying impacts on toxin cycling and health. Dose-effect and toxin cycling elucidation is challenging in environmentally exposed wildlife due to opportunistic testing, rapid DA elimination, and unknown consumption dose, duration, frequency, history, and time elapse between DA ingestion and work-up. Thus, the toxicokinetic properties of DA after environmental exposure are presumptive.

Humans and marine wildlife may be exposed to DA via the ingestion of contaminated zooplankton, shellfish, finfish, or seawater, or through maternal toxin transfer [89]. Prey that bioaccumulate DA demonstrate markedly different elimination rates from days in finfish to years in some shellfish [82,83,84]. In nonhuman primate and murine models, DA is slowly and poorly absorbed from the GI tract and diffuses poorly through the placenta and blood-brain barrier (BBB) [90,91,92]. The toxin can bioaccumulate in amniotic fluid and breast milk for weeks, magnifying perinatal exposure [18,93,94,95,96]. Access to the CNS may be augmented by an increased BBB permeability from DA-induced seizures or via the less protected circumventricular organs [26,97]. Due to DA’s high potency, small quantities may be capable of extreme toxicity. Active transporters for DA distribution into body compartments may be present in some species [92,98].

Domoic acid primarily exerts its toxic effects through excessive stimulation of GluRs, resulting in cell death and organ dysfunction [99]. Mammalian GluRs are categorized into two families: (1) ionotropic (iGluRs; fast-acting ligand-gated cation channels responsible for excitatory neurotransmission) and (2) metabotropic (mGluRs; slower-acting G-protein-coupled receptors that modulate excitatory neurotransmission) [81]. Since DA primarily interacts with iGluRs, toxicity is most substantial in cells and body regions (e.g., the hippocampus) where they are highly concentrated. Neural and extra-neural tissues (e.g., heart, kidneys, liver, lungs, reproductive organs, adrenal and pituitary glands, and GI tract) contain iGluRs and may experience toxicity [39,94,95,96,97,98]. Domoic acid may also interact to a lesser degree with mGluRs [100,101]. In addition to direct activation of local GluRs, toxicity in peripheral tissues (e.g., heart and GI tract) may be due to central activation [97,100,102,103,104,105].

Domoic acid induces a complicated cascade of cellular events depending on the exposure scenario. In acute high-dose exposures, DA stimulates all CNS iGluR subtypes, causing elevated intracellular calcium levels, reactive oxygen species (ROS) production, and subsequent necrosis, whereas low-level exposures likely largely involve apoptosis without all iGluR subtypes or ROS [99,106,107]. Acute toxicosis, followed by an asymptomatic latency period with structural and functional damage, culminating in progressive disease, also occurs [30,108]. Other mechanisms of CNS or peripheral injury include the following: (1) osmotic tissue damage, (2) mitochondrial disruption, (3) inadequate neuroprotection provided by astrocytes, (4) inflammation, (5) dysregulation of gamma-aminobutyric acid (GABA, the primary inhibitory CNS neurotransmitter), (6) immune imbalance, (7) gene expression changes, (8) altered endocrine homeostasis, (9) vascular damage, and (10) cardiac conducting system dysfunction [41,106,108,109,110,111,112,113,114,115,116,117].

Domoic acid is eliminated from the body unchanged, suggesting limited metabolism [26]. The kidneys serve as the primary systemic elimination route [88,98,118,119]. Preferential renal accumulation with equal levels in the medulla and cortex, glomerular filtration with active tubular resorption, tubular secretion using organic anion transport, biliary secretion, and cerebral spinal fluid (CSF) elimination have been observed [25,88,90,92,95,118,119,120,121]. While depuration rates vary among impacted species, from hours in CSLs to over a week in humans, clearance is generally considered rapid [122,123]. Elimination may be delayed in dehydrated individuals [124].

A dose-effect continuum occurs where lower DA doses cause acute GI upset, higher doses lead to acute neurotoxicity or death [102], and long-term, low-level exposure can result in subtle neurobehavioral deficits [125]. A DA dose-response estimate for acute toxicosis and death in a common minke whale (*Balaenoptera acutorostrata*; 1.1–6.8 mg/kg) was consistent with the estimated lethal dose in humans (4 mg/kg) [24,27,89]. Exposure dose estimates based on prey consumption suggest that doses below 1.1 mg/kg are not overtly neurotoxic in mysticetes [44,126]. Pre-conditioning permitted a neuroprotective tolerance to DA in the hippocampus of young, but not aged, rats in situ [127], whereas enhanced neurologic sensitivity was observed after intracoelomic DA administration in finfish models of repeated exposure [128]. A potential adaptive response was observed in the CNS of nonhuman primate models of long-term oral DA exposure [129]. These studies highlight the potential contribution of exposure history, exposure route, and species to dose-response relationships.

## 5. Susceptibility

Biological and environmental factors affect susceptibility to DA toxicosis, including individual (i.e., species, age class, sex, underlying health status, and exposure history) and population-level (i.e., home range, prey availability, and conservation status) influences.

### 5.1. Species

Humans are one of the most DA-sensitive taxa due to physiologic, geographic, and socioeconomic factors. Groups at an increased risk based on seafood consumption rates include (1) coastal residents [62], (2) commercial or recreational anglers [130], (3) individuals who perceive health benefits from, and can afford, omega-3-rich seafood, and (4) indigenous communities who rely on shellfish for culture and nutrition [131]. Domoic acid susceptibility may be mitigated through seafood monitoring programs in humans and piscivorous wildlife under human-managed care. Long-lived taxa, such as humans and marine mammals, are more susceptible to repetitive exposure or likely to experience delayed or progressive impacts [132]. Nonhuman primate models may be most translatable to human DA sensitivity [133].

Though DA has been detected in a range of marine fauna, from zooplankton to large whales [44], linking exposure to disease susceptibility is often challenging due to inaccessible open-ocean-dwelling species, unknown mechanisms of action, and subtle or nonspecific lesions. Marine mammals have enhanced susceptibility to DA-induced systemic impacts due to their anatomy and physiology, diet, and foraging characteristics. During diving, blood is shunted to the most vital, yet DA-sensitive organs (i.e., CNS and heart) and restricted to those responsible for elimination (i.e., GI tract and kidneys) [134]. Marine mammals are exposed to a larger DA dose per body weight due to the hydrophilic toxins’ poor distribution to blubber [89]. Southern sea otters are uniquely susceptible because of their high daily dietary intake of primarily marine invertebrates, which biomagnify and slowly depurate DA [41]. Avians may be susceptible across taxa since seabirds and landbirds have demonstrated environmental and experimental DA toxicosis, respectively [135]. However, marine birds have an enhanced risk of environmental exposure. Marine megafauna with DA exposure and/or individual or population-level impacts are summarized (Table 1).

Domoic acid toxicosis from environmental exposure has not been definitively diagnosed in finfish, elasmobranchs, or invertebrates. However, mortality concurrent with DA-producing blooms, as well as DA toxicosis after experimental administration, have been observed in these species [2,35,152,153,154,155,156]. Moreover, DA can accumulate in their neural and extra-neural tissues, which also possess GluRs [87,152,153,154,155,156]. These taxa may avoid DA toxicosis from environmental exposure due to molecular mechanisms [157] or rapid toxin depuration [88], or they may experience subclinical disease [152]. Experimental DA administration caused developmental anomalies and death in finfish [155,158], as well as genotoxicity, immunomodulation, oxidative stress, respiratory dysfunction, and developmental and neurobehavioral disease in marine invertebrates [121,126,154,159,160,161,162,163,164,165,166,167,168,169,170,171,172]. Along with the toxins’ historical use as an anthelmintic, these studies underscore the potential for sensitivity and subsequent impacts on the fitness and survival of free-ranging marine finfish, elasmobranchs, and invertebrates.

### 5.2. Age

Age-related DA sensitivity and health effects vary depending on taxa and exposure scenarios. Age may influence DA resistance, as young, but not aged, murine developed a preconditioned tolerance in the CNS in situ [127]. Developing and aged individuals are generally the most sensitive. Susceptibility and health impacts due to environmental DA exposure should be evaluated over the lifespan [173,174], particularly in long-lived species.

The increased DA susceptibility in developing and aged individuals is primarily due to less effective physiological protections. Renal clearance and BBB integrity are reduced [106,175]. Toxin recirculation via the amniotic fluid can further amplify prenatal exposure dose and duration [93]. Windows of increased susceptibility and impacts of exposure are based on species-specific milestones, such as the developmental stage (e.g., neurogenesis, neuromigration, or synaptogenesis) during which exposure occurs [125,173,175,176]. Most species have an enhanced susceptibility during early gestation due to the intensity/stage of neurodevelopment. Perinatal CSLs are more vulnerable in certain regions due to the spatiotemporal overlap of blooms, the pupping season, and maternal foraging areas [175]. Decreased levels of neuroprotective hormones and antioxidants may contribute to the increased sensitivity in aged individuals [177,178,179]. Health impacts associated with an enhanced DA sensitivity in these age groups include (1) a higher incidence of seizures and hospitalization, greater death rates, and prolonged neurologic deficits in aged ASP patients [24]; (2) delayed and permanent neurobehavioral disease, socioemotional deficits, and cardiac and spinal cord defects, as well as altered concentrations of CNS neurotransmitters and their receptors after developmental exposure [125,132,158,174,180,181]; (3) an increased incidence of domoic acid-induced epileptic disease in younger CSLs [108]; and (4) decreased survival rates in CSLs exposed in utero [17,182].

Adults may experience enhanced DA susceptibility under some conditions. The spatiotemporal overlap of foraging sites with toxigenic blooms results in an increased risk to some mature cetaceans and pinnipeds [11,93,139]. For this reason, adult female CSLs and northern fur seals (NFSs, *Callorhinus ursinus*) in California are at the highest risk of developing acute toxicosis [108,139]. Physiology may also contribute to prime-age DA sensitivity, as evidenced in some model species [183,184], as well as by increased severity of brain damage and risk of cardiomyopathy in marine mammals [185,186].

### 5.3. Sex

Marine wildlife exhibit population-specific sex predilections where males or females are more at risk for acute toxicosis based on DA loads in preferred prey and foraging locations, as well as unknown factors. Regional sex predilections to acute DA toxicosis include mature male CSLs in Baja, Mexico [187], mature female CSLs in central California (predilection of 24:1) [108,188], female NFSs in California [139], and male common dolphins in California [11,43]. Sex-related feeding niche segregation, as well as a wider isotopic niche, may have contributed to a higher risk of male CSLs in Baja, Mexico [187]. Conversely, male CSLs in central California are fasting while breeding during their temporary occupancy in areas highly impacted by DA-producing blooms prior to migrating to less impacted regions [108]. The higher risk to female CSLs and NFSs in California is attributed to foraging near breeding rookeries with high DA loads [108,139]. Male common dolphins often forage in bachelor pods that may target highly impacted areas or prey items [11,43]. For example, DA load was associated with higher consumption of an efficient DA vector, the northern anchovy (*Engraulis mordax*), in male common dolphins compared to females [11]. Male brown pelicans (*Pelicanus occidentalis*) may also have an increased susceptibility to acute toxicosis, but the potential cause is unknown [34]. No sex predilection is apparent with domoic acid-induced epileptic disease or chronic toxicosis in wildlife [108].

Sex-based sensitivity varies in humans and laboratory models based on organ systems, exposure scenarios, and hormonal fluctuations during the lifespan [177,179]. Males tend to be more sensitive to DA-induced neurobehavioral impacts [189,190], while females may have an enhanced susceptibility to acute renal damage [191]. The most seriously ill ASP patients were male, possibly due to an increased exposure dose, more compromising underlying health conditions, or socio-emotional or biological influences [26]. Male rodents are most sensitive to long-term, low-level DA exposure [192], and there are sex-specific differences in socioemotional impacts in developing individuals [190,193,194,195,196].

### 5.4. Underlying Health Status

Co-morbidities, especially those affecting vital organs or tissues with high concentrations of iGluRs, may increase an individual’s DA sensitivity. Pre-existing conditions, including insulin-dependent diabetes, chronic renal disease, hypertension, autoimmune disease, chronic liver dysfunction, pituitary adenoma, and Parkinson’s disease (PD), were associated with an increased ASP severity [197,198], partly due to less effective toxin neutralization or clearance. Humans and marine wildlife commonly experience kidney or cardiac dysfunction [199,200,201,202], thus amplifying their vulnerability to DA exposure. Gastrointestinal lesions were hypothesized as a predisposing factor to DA toxicosis related to gut absorption kinetics [97]. Though GI ulcers are common in CSLs and other marine wildlife [201], there are no published studies to date to support this hypothesis. Metabolic, hormonal, cardiovascular, and immune changes during pregnancy may increase maternal DA sensitivity [93,203].

### 5.5. Exposure Scenario

Extrinsic factors influence individual or population-level DA susceptibility. An individual’s exposure history, including DA dose, frequency, and duration, may influence sensitivity [128,129]. Previous DA exposure may lead to a decreased or enhanced neurologic sensitivity [127,128], or an adapted CNS response [129]. Concurrent exposure to other biotoxins or contaminants may increase vulnerability to DA [15]. Factors that may affect population-level DA susceptibility include toxin bioaccumulation kinetics in preferred prey, prey availability, and the spatiotemporal overlap between toxigenic blooms, vector prey, and the impacted species’ home range [204,205]. For example, anchovies are abundant planktivores that are efficient DA vectors and dominate over sardines during certain climate cycles off coastal California [2]. Domoic acid exposure may have greater impacts on the stability of endangered (e.g., northern right whales, green sea turtles, and leatherback sea turtles), threatened (e.g., SSOs, Guadalupe fur seals (*Arctocephalus Townsendi*), ringed seals (*Phoca hispida*), bearded seals (*Erignathus barbatus*)), and keystone (e.g., SSOs) populations, as well as those in decline (e.g., CSLs in the Gulf of California and Scottish harbor seals (*Phoca vitulina*)) [44,45,191,192,193].

## 6. Clinical Signs and Symptoms

Due to the broad tissue distribution of GluRs, DA may impact numerous organ systems. Clinical signs are commensurate with the organ affected, range from subtle to conspicuous and non-specific to specific, are dependent on exposure dose and time lapse since exposure, and may go unobserved in marine wildlife due to a delayed presentation to health managers.

### 6.1. Gastrointestinal

Domoic acid-induced GI symptoms in marine wildlife and humans include abdominal cramps, hypersalivation, gagging, retching, lip licking/smacking, regurgitation, nausea, vomiting, diarrhea, inappetence, gastric bleeding, and fecal impaction [24,34,41]. The most common symptoms in ASP patients were GI-related [24]. Domoic acid may induce vomiting and regurgitation acutely in seabirds [34], fecal impaction, inappetence, and nausea acutely to chronically in SSOs [41], and vomiting and inappetence chronically in CSLs [106].

### 6.2. Central Nervous System

The most common DA-induced CNS signs across species and exposure scenarios are seizures and/or cognitive deficits. Hallmark CNS signs include long-term amnesia in ASP patients, as well as hindlimb scratching and atypical aggression in wildlife [24,41,108]. Most CNS signs are from limbic system injury, but damage to other areas can also manifest in symptoms [204].

In ASP patients, CNS signs primarily occurred within 48 h and included mutism, purposeless chewing and grimacing, emotional lability, severe headache, visual disturbance, dizziness, confusion, anterograde and/or retrograde amnesia, hallucinations, cranial nerve deficits, lack of response to painful stimuli, seizures, posturing, myoclonus, generalized weakness, unsteadiness, and coma [24,197]. While these were typically reversible, permanent anterograde amnesia occurred in some patients, and another developed TLE one year after recovery [24,30]. Long-term, low-level DA ingestion caused transient or permanent memory loss depending on the exposure dose in some members of Indigenous communities in the Pacific Northwest, USA [18,31,32,206,207].

Only acute CNS signs have been reported in seabirds, and these include abnormal posturing, inability to take flight or retract legs during flight, fine motor tremors, head weaving, scratching, disorientation, loss of awareness or righting reflex, ataxia, toe clenching, paddling, weakness, paralysis, and anomalous behaviors (e.g., unusually agitated or docile and asocial behavior) [34,37,135]. California sea lions with acute toxicosis often strand in clusters, while those with the chronic disease following toxicosis are more likely to strand individually and in atypical locations [17]. Central nervous system symptoms most commonly associated with acute toxicosis in CSLs include ataxia, head weaving, and scratching, seizures, and coma [40], whereas those more commonly associated with chronic disease include spontaneous, intermittent, and progressive seizures, muscle twitching, periodic lethargy, anomalous behaviors (e.g., conspecific or human-directed aggression, frantic pacing, rocking, circular swimming, backward circular walking, and chewing), central blindness, blepharospasm, spatial memory deficits, delayed habituation, enhanced dishabituation, and foraging difficulty in the wild due to behavioral inflexibility [17,108,132]. Similar neurobehavioral signs are observed in other pinnipeds and fissipeds with DA toxicosis [137,139,140], but these diminish in favor of cardiac signs in SSOs with chronic disease [41]. Other CNS signs in SSOs include hyphema, trouble with ambulation or food prehension acutely, paresthesia, hindlimb paraparesis, fecal impaction, and urine retention chronically [41].

Laboratory models of DA toxicosis can demonstrate subtle or long-term CNS impacts that are difficult to ascertain after environmental exposure. Examples include reversible memory and learning deficits after a wash-out period from long-term exposure [181], a reduced seizure threshold in adulthood after low-dose, perinatal exposure [208], and intention tremors from long-term, low-level exposure during pregnancy [125]. These findings may have implications for the well-being of environmentally exposed species.

### 6.3. Cardiovascular/Respiratory

Domoic acid-induced cardiorespiratory signs include (1) heart palpations and dyspnea in ASP patients and (2) dyspnea, cyanosis, oronasal froth, heart murmur, and pulse deficits in SSOs and CSLs [24,26,41,104,209]. Cardiac signs typically predominate in SSOs with subacute to chronic DA toxicosis.

### 6.4. Urogenital

The most prominent DA-induced urogenital symptom is reproductive failure in CSLs and SSOs with acute toxicosis, manifesting as abortion, stillbirth, premature parturition, or death of pregnant dams [41,93]. Male SSOs with subacute to chronic toxicosis may have testicular atrophy [41], which could impact reproductive success through reduced sperm production and quality, as well as lower testosterone levels.

### 6.5. Integumentary/Musculoskeletal

While marine mammals with acute DA toxicosis typically demonstrate normal body condition, those with chronic toxicosis may be emaciated due to reduced foraging success [40,41,47,89]. Southern sea otters with acute toxicosis usually have healthy pelages (except for peri-oronasal staining), whereas those with subacute to chronic disease may have poorly groomed, dry, and/or traumatized (fight- or self-induced) pelages [41]. Piloerection was observed in some ASP patients [197].

### 6.6. Other/Indirect

Clinical signs may be indirectly triggered by DA blooms. For example, the “Warm Water Anomaly” caused marine mammal habitat and prey shifts and fisheries closures, inadvertently causing an unprecedented number of large whale entanglements [210]. Similarly, DA-producing blooms were associated with injury and death from fishing gear entanglement and vessel strikes in large whales [51].

## 7. Diagnostic Tests

This section will review pre- and post-mortem diagnostic tests for confirming DA exposure and related disease. Few tests have been assessed for specificity or sensitivity or are routinely utilized after environmental exposure [209]. Appropriate test selection is determined by available sample(s), resources, species, and exposure scenario. No single test confirms DA toxicosis; thus, methods must be used in tandem. A combination of environmental and patient data is often required, along with a “weight of evidence” approach [211]. Definitive confirmation is complicated by the rapid clearance rate of the toxin in many species, delayed manifestations of disease, subtle or non-specific clinical signs and pathologies, inadequate resources for sample collection or testing, and unknown clinical or exposure histories.

### 7.1. Diagnosis of DA Exposure

To diagnose acute DA exposure, the toxin and/or toxigenic diatom should be detected in water samples in spatiotemporal proximity to a marine wildlife stranding event, as well as within vector prey and the impacted individual [204]. This may be infeasible in cases of subacute to chronic toxicosis with delayed disease manifestations [211].

#### 7.1.1. *Pseudo-nitzschia* spp. Detection

Seawater or prey samples in known habitats of intoxicated individuals, as well as GI contents, should be assessed for *Pseudo-nitzschia* spp. frustules. A false negative result may occur if the diatom or vector prey are no longer present in the environment or GI tract at the time of clinical presentation, with cryptic blooms or limited monitoring capabilities. The presence of environmental diatoms does not guarantee exposure, and diatoms may not be toxigenic [140]. Light microscopy is typically insufficient for species identification. Scanning electron microscopy, transition electron microscopy, or molecular techniques should be used for taxonomic delineation [1,204]. Molecular techniques can distinguish between diatom species that are morphologically comparable and may also be useful for species quantification [1,212].

#### 7.1.2. Domoic Acid Antigen Detection

Detection of DA is vital, when possible, to help confirm disease caused by acute exposure but can also support the diagnosis of subacute to chronic toxicosis if data is available regarding previous DA exposure in an individual. Matrices for DA antigen detection include diatom, seawater, or prey samples and/or body fluids and tissues (i.e., GI/cloacal contents, feces, urine, serum, bile, breast milk, kidney, liver, brain, aqueous humor, pericardial fluid, CSF, amniotic fluid, fetal meconium, and allantoic fluid) of impacted individuals [25,39,96,139]. Serum, feces, and urine are usually the most readily available and accurate pre-mortem matrices [123,213]. Measured DA antigen levels may not accurately represent the exposure dose due to toxin depuration and unknown baseline body burdens [128,214,215]. Enzyme immunoassay (ELISA) and/or liquid chromatography-tandem mass spectrometry (LC-MS/MS) are commonly used alone or jointly [123,216]. While ELISA is rapid, sensitive, and easily accessible, some yield an indirect measurement that can be skewed by isomers, DA antibodies, or matrix interference [213,214]. Alternatively, LC-MS/MS provides a direct measurement that is highly sensitive and specific but requires a substantially greater time and resource investment [213]. These tests may require toxin extraction to help reduce matrix effects despite an increased DA recovery variability. An appropriate internal standard can mitigate test limitations [213].

#### 7.1.3. Domoic Acid Antibody Detection

Serum DA antibodies have been detected in CSLs with acute toxicosis immediately and up to several weeks later via ELISA, as well as in humans with previous or long-term exposure via ELISA and surface plasmon resonance (SPR) biosensor [123,128]. Though SPR had high sensitivity and specificity [123], the development of clinical DA antibody tests was halted due to logistical constraints (K. Lefebvre, personal communication, October 2021).

#### 7.1.4. Vector Prey Detection

Vector prey are specific to the region and species impacted. Their identification and quantification (e.g., via otolith examination) within the GI tract of impacted individuals can explain the exposure pathway and aid exposure dose calculations [44,89,126,152].

### 7.2. Diagnosis of DA Toxicosis

There are many potential tests for diagnosing DA toxicosis, including generalized, organ-specific, and emerging methods. Findings vary based on the exposure scenario and species. Unlike the detection of the DA antigen, very few current diagnostic tests are specific for DA toxicosis.

#### 7.2.1. Blood Parameters

Serial complete blood count (CBC) and serum biochemistry tests can evaluate for DA-induced systemic impacts, assess longitudinal health trends, and detect delayed effects. Anomalies after environmental exposure can be inconsistent and are nonspecific but may indicate immunomodulation, dehydration, and kidney or muscle damage (Table 2). Examples of DA-induced immunomodulation are leukocytosis in ASP patients and dolphins with subclinical exposure [26,67,145], lymphocytopenia and monocytosis in seals with subclinical exposure [141], and eosinophilia in CSLs and dolphins with acute or chronic toxicosis [67,145,217]. Elevated levels of creatine phosphokinase (CPK)/creatine kinase (CK) are observed in several species with acute toxicosis due to seizure-induced muscle damage [26,34,40]. Abnormal serum creatinine (sCr), blood urea nitrogen (BUN), uric acid (UA), and/or hematocrit (HCT) may be due to dehydration or kidney damage with altered water reabsorption and red blood cell (RBC) production [26,34,40,137,197,209]. Immune suppression or modulation, as well as altered hemolymph biochemical parameters (i.e., pH, bicarbonate, alkaline phosphatase (ALP), lactate dehydrogenase (LDH), and total protein (TP)), have been observed in DA-exposed marine invertebrates [159,160,162,170].

#### 7.2.2. Hormone Testing

Cortisol and thyroid hormone levels may be altered by DA exposure. Serum cortisol levels may be low in CSLs with acute or chronic toxicosis [217]; fecal glucocorticoid metabolites (fGCm) negatively correlate with exposure in southern right whales [219], and fecal cortisol positively associates with exposure in northern right whales [47]. Though serum thyroid hormone (i.e., T3, T4) and thyroid-stimulating hormone (TSH) alterations were observed in models of DA toxicosis [220,221], as well as the decreased transcription of a thyroid hormone receptor in northern sea otters (*Enhydra lutris kenyoni*) with suspect repetitive, low-level DA exposure [222], thyroid hormone impacts from environmental exposure require investigation. Adrenal and thyroid gland ultrasonography can evaluate for DA-induced disease in the tissues responsible for hormone production and/or release.

#### 7.2.3. Kidney Injury Biomarkers

Serum kidney function markers (e.g., BUN and sCr) may have limited use in diagnosing DA-induced acute kidney injury [119] but may aid in monitoring the development and progression of dysfunction over time. Elevated levels of urinary markers of acute tubular injury, neutrophil gelatinase-associated lipocalin (uNGAL) and kidney injury molecule-1 (uKIM-1), occurred 72 h after DA exposure in murine models [119], but their use, along with renal ultrasonography, after environmental exposure requires investigation.

#### 7.2.4. Cardiovascular Injury Biomarkers

Tests for DA-induced cardiac injury include serum biomarkers of heart damage, chest imaging, electrocardiogram (ECG), and blood pressure measurement. Modalities may require expertise for sedation/general anesthesia and/or interpretation in wildlife, which could impact results.

Serum troponin-1 (cTn1) and N-terminal pro-brain natriuretic peptide (NT-proBNP) were validated as cardiac injury biomarkers in several marine mammal species [223]. Though CSLs with DA toxicosis may have considerable cTnI elevations, single measurements are not predictive of cardiomyopathy and cannot differentiate acute vs. chronic disease, possibly due to cTn1’s short plasma half-life [104]. Serial cTn1 levels can help diagnose and monitor DA-induced cardiac disease in SSOs [224] and may be useful in other species. While skeletal and cardiac muscle L-carnitine are not predictive of DA-induced cardiac disease in CSLs, skeletal levels may help differentiate acute vs. chronic disease [104].

Imaging of the chest cavity may allow diagnosis and monitoring of DA-induced cardiomyopathy and subsequent congestive heart failure. Echocardiographic anomalies in CSLs with DA toxicosis include reductions in cardiac output, fractional shortening, and ejection fraction, as well as valvular insufficiency and abnormal septal wall movement [209]. Transthoracic echocardiography can be challenging in sea otters due to their dense and vital pelage [225]. Serial chest radiographs can help assess DA-induced cardiac disease in SSOs and may be valuable in other species [186].

Domoic acid-induced ECG anomalies include a decreased P wave amplitude, atrial and ventricular ectopy, first or second-degree atrioventricular (AV) block, and a sinoventricular rhythm in SSOs with subacute to chronic toxicosis [224], as well as 1st or 3rd degree AV block and premature atrial and ventricular complexes in CSLs [209]. Tracings do not correlate with disease in CSLs and, thus, may not accurately detect cardiac injury [201]. Continuous ECG and blood pressure measurements are recommended in ASP patients to monitor for arrhythmias and hypotension [24,198].

#### 7.2.5. Neurobehavioral Injury Markers

Electrography, advanced imaging, and neurobehavioral tests can support the diagnosis of DA-induced CNS disease, as well as assess the type, localization, and severity of damage.

Electroencephalograms (EEGs) and electromyograms (EMGs) can evaluate CNS and muscle electrical activity, respectively. Nonspecific EEG anomalies in ASP patients included generalized slowing of background activity and epileptogenic activity in the frontotemporal region in the acute phase, as well as generalized disturbance of background activity in the temporal lobes and periodic epileptiform discharges in the chronic phase [24,25,30]. In CSLs, EEG findings include epileptiform discharges characterized by spikes, sharp waves, slow waves, and/or spike waves of varying severity and localization [226], as well as intermittent rhythmic delta activity often followed by background attenuation localized to the posterior cerebral hemispheres with chronic toxicosis [17,108]. However, these findings cannot differentiate between acute and chronic cases and may be observed with other etiologies [17,108,226]. In ASP patients, EMG anomalies a few months after exposure included spontaneous activity, neurogenic recruitment, and unstable motor unit potentials [25,26]. Anomalous EEGs and EMGs in models of DA toxicosis may have relevance to environmental exposure [227,228].

Magnetic resonance imaging (MRI) and positron emission tomography (PET) scans can help diagnose DA-induced CNS disease. Anomalies are primarily localized to the limbic system, specifically the hippocampus, amygdala, and parahippocampal gyrus. Neuron loss and decreased glucose metabolism were observed in ASP cases [197,229] vs. bilateral atrophy and glucose metabolic deficits in the TLE patient [30]. Anomalies in CSLs with DA toxicosis can be unilateral to bilateral and progressive and include structural loss/atrophy, mossy fiber sprouting, altered connectivity, pathologic T2 hyperintensity, gliosis, decreased glucose metabolism, neuropil neocapillarization, and temporal horn enlargement [17,106,132,185,230,231,232,233]. Regional encephalitis and swelling that progressed to atrophy were observed in a Guadalupe fur seal with chronic toxicosis [137]. Imaging anomalies may be absent in the acute phase even with severe symptoms and tissue damage [101]. Long-term DA administration in nonhuman primates caused decreased white matter integrity in hippocampal motor tracts and increased brain lactate [133], but findings may not extrapolate to environmental exposure.

Neurobehavioral tests may aid in diagnosing DA toxicosis and related deficits. Patients with ASP and individuals who developed TLE exhibited memory deficits, whereas language function, concept formation, and verbal comprehension were normal [30,197,234]. Decreased cognition and memory in CoASTAL cohort participants was likely due to repetitive, low-dose DA exposure [18,32,206,207]. Tests in CSLs with DA toxicosis include (1) behavioral ethograms, in which head weaving, muscle fasciculations, hind flipper dragging, and swift scanning are diagnostic for acute or chronic disease [124], (2) auditory responsive behavioral tests, in which delayed habituation can help diagnose acute or chronic disease [132,235], (3) spatial memory assays, in which performance may positively correlate with the dorsal right hippocampal volume on MRI [230], (4) “clinical assessment scores” and “neuroscores/(NS)”, which negatively correlate with prognosis [40,209], and (5) olfactory function testing, which may identify impairment in chronic disease [236]. Studies in models of acute, long-term, or developmental DA exposure demonstrated impairment of learning, memory, cognition, passive avoidance, startle response, motor coordination, conditioned avoidance response, circadian activity, gait, habituation, and exploratory behavior [125,176,181,190,237,238,239,240,241], which may extrapolate to environmental exposure.

#### 7.2.6. Reproductive Failure Confirmation

Abdominal ultrasound and/or radiographs can assess for pregnancy, fetus viability, and DA-induced reproductive disease (i.e., uterine torsion/rupture or testicular atrophy) [209].

#### 7.2.7. Gastrointestinal Lesion Identification

Abdominal ultrasound, endoscopy, and/or radiographs can evaluate for DA-induced GI ulceration, torsion, and intussusception.

### 7.3. Gross and Microscopic Histopathology

Gross and microscopic histopathology are performed post-mortem due to the invasiveness of pre-mortem collection of the primary organs impacted. Microscopic histopathology is often the gold standard for diagnosing DA toxicosis. Gross pathology can be distinctive but is frequently absent. Limited pathology may be present even with substantial clinical signs, particularly in seabirds and cetaceans [34,146]. Most histopathologic scrutiny focuses on the CNS, but peripheral organs can also suffer damage. Specific immunostains (e.g., neurodegeneration biomarkers) can support histopathologic findings [242].

#### 7.3.1. Gastrointestinal Tract

On gross exam, GI tracts may be empty due to nausea, inappetence, or vomiting, and erosions, ulcers, or bleeding may be present [24,41,101]. Findings in SSOs include full GI tracts acutely, atrophic empty tracts chronically, GI torsion, ileus, or intussusception acutely to subacutely, and congestion [41].

#### 7.3.2. Central Nervous System

There are minor differences in the type, distribution, and severity of DA-induced CNS lesions based on species, exposure scenario, and time lapse since exposure. Damage to limbic structures is most common and severe, particularly the hippocampal pyramidal neurons in CA1, CA3, and CA4 sectors and the dentate gyrus granule cells. Other impacted regions may include the amygdala, thalamus, hypothalamus, subfrontal cortex, medulla oblongata, pons, cerebellum, piriform and entorhinal cortices, olfactory bulbs and tracts, septum, nucleus accumbens, arcuate nucleus, circumventricular organs, ependyma, cingulate gyrus, occipital lobe, choroid plexus, ventricles, pituitary, retina, and spinal cord [25,30,41,108,137,222]. Lesion symmetry is typically bilateral in ASP cases and unilateral or bilateral in pinnipeds and fissipeds [17,41,243].

The most prominent CNS lesions in ASP cases were neuronal necrosis and sclerosis in the amygdala, hippocampal pyramidal neurons, and dentate gyrus [25], which progressed to hippocampal atrophy, ventricular dilation, and neuronal loss and gliosis of all CA sectors, the amygdala, and secondary olfactory areas in the TLE patient [30]. While CSLs with acute toxicosis lack gross CNS lesions, parahippocampal and hippocampal atrophy and lateral ventricle dilation may be observed in chronic cases [101]. Microscopic lesions include (1) per-acute microvesicular hydropic degeneration in the neuropil of limbic structures and laminar vacuolization in CA3 pyramidal cells, (2) acute ischemic neural necrosis in the hippocampal formation, and (3) chronic neuronal loss, atrophy, gliosis, non-suppurative inflammation, laminar disorganization, sclerosis, and mossy fiber sprouting predominately in the hippocampus and parahippocampal gyrus [17,101,108,244]. Focal meningeal hemorrhage and brain edema in parahippocampal areas are also observed in immature CSLs with chronic toxicosis [17]. Brain edema may be present in pinniped pups exposed in utero [140,182]. Compared to humans, CSLs exhibit more frequent dentate gyrus and asymmetric injury, as well as less damage in sector CA1 [17]. Other pinnipeds have similar CNS lesions as CSLs, though time-dependent patterns are not established. Differences include less common perivascular cuffing with lymphocytes in NFSs [139] and CA2 involvement in Pacific harbor seals [139,140], as well as prominent cerebellar folia lesions in a Guadalupe fur seal [137].

Southern sea otters with acute toxicosis primarily demonstrate gross congestion and hemorrhage of the meninges, neuropil, and ventricles, as well as pink discoloration of the neuropil, whereas hippocampal atrophy, ventricular enlargement, and pale tan neuropil predominate in chronic cases [41]. Instead of the nerve and glial cell necrosis and degeneration observed acutely in other species, SSOs demonstrate congestion and microhemorrhages in the brain, spinal cord, and meninges. Pituitary gland lesions are a distinguishing feature of acute disease in SSOs. Those with chronic toxicosis typically demonstrate neuronal and glial cell loss with necrosis, apoptosis, gliosis, spongiosis, scarring, and “moth-eaten” cells, and SSOs more frequently experience severe damage in the CA2 sector and spinal cord involvement than other species [41].

Seabirds are the only other wildlife with documented DA-induced CNS damage, which includes acute neuronal necrosis with capillary endothelial cell hyperplasia [34].

#### 7.3.3. Ocular

Domoic acid-induced retinal damage is in the ganglion cell layer in CSLs vs. the inner nuclear and outer plexiform layer in model species [26,101,102,245]. Fibrinous ophthalmitis may be present in CSLs with acute disease [101]. Congestion and microhemorrhages in the choroid and ciliary body, as well as hyphema, may aid the diagnosis of acute toxicosis in SSOs [41].

#### 7.3.4. Cardiovascular/Respiratory

The following lesions are observed in SSOs with DA-induced degenerative cardiomyopathy: (1) gross pulmonary hypersecretion and evidence of congestive heart failure, such as discolored myocardium, hemorrhage, venodilation, congestion, venous shunts, cardiomegaly, and pericardial, pleural, peritoneal, or pulmonary edema and fibrosis, and (2) microscopic acute myocardial and vascular congestion, microhemorrhages, necrosis, apoptosis, edema, and vacuolation [41]. Subacute to chronic lesions are more severe, advanced, likely to involve non-suppurative inflammation, and predominated by cardiomyocyte loss, fatty replacement, fibrosis, and coronary arteriosclerosis [41]. Pinnipeds with DA toxicosis may have similar cardiac lesions as SSOs, as well as bronchopneumonia [104,139,140], but lesion progression is not described. Domoic acid-induced cardiac lesions are presumptive in cetaceans [246], have not been described in ASP patients, and are predominated by focal myofiber necrosis in seabirds [34].

#### 7.3.5. Urogenital

Urinary tract anomalies from environmental DA exposure include kidney edema/congestion in SSOs and CSLs with acute to chronic disease, renal tubular regeneration in CSLs with acute disease, and bladder distention in SSOs with acute to chronic disease [40,41,187]. Proximal tubular necrosis with vascular and tubular damage, cytoplasmic eosinophilia, cell desquamation, mitochondrial swelling, and vacuolization were observed in the kidneys of rodent models of acute toxicosis [119].

Gross genital tract lesions in pinnipeds and SSOs include abortion, fetal resorption, stillborn or preterm fetus, congestion/edema of the uterus or placenta, uterine torsion, prolapse, or rupture, and signs of forced copulation outside of estrus [41,93]. Microscopic lesions include hyper-eosinophilia, edema, hemorrhage, apoptosis, or necrosis within or around the myometrium, myometrial vasculature, and placenta, as well as testicular atrophy in SSOs with subacute to chronic disease. Fetal pathology resembling adult lesions can lead to abnormal growth, development, and survival [164,165].

#### 7.3.6. Integumentary/Musculoskeletal

Gross anomalies include focal hemorrhages in the forelimb and hindlimb musculature of seabirds with acute toxicosis [34], as well as a dry unhealthy pelage, decreased fat/blubber stores, cachexia, and skeletal muscle or serous atrophy in marine mammals with chronic toxicosis [41,47,101]. Seabirds may demonstrate focal to multifocal skeletal muscle necrosis microscopically [34].

#### 7.3.7. Other

Damage to other organs occurs with less consistency or severity, such as vascular congestion in the adrenal glands and progressive congestion, hemosiderosis, and necrosis in the liver [41]. It is unclear whether these lesions correlate with clinical signs.

### 7.4. Molecular Diagnostics

Molecular diagnostics can elucidate (1) mechanisms of DA-induced tissue injury [119,247], (2) biomarkers of exposure [248,249,250,251], and (3) differential diagnoses (e.g., acute vs. chronic DA toxicosis vs. other) [248,252]. These tests require specialized equipment and expertise and, thus, may not be feasible in the clinical setting. Some methods only permit analysis of a limited number of proteins or genes, though others allow global evaluation. While many studies used molecular diagnostics in models of DA toxicosis [121,128,129,158,169,170,172,241,253,254,255], Table 3 summarizes findings after environmental exposure.

## 8. Time Course of DA Toxicosis

A range of time-dependent processes are possible after DA exposure, including per-acute, acute, subacute, chronic, and acute-on-chronic disease [209]. These can be difficult to differentiate due to a disease spectrum based on DA exposure dose and history. Criteria for delineating these processes are incomplete and only available for SSOs and CSLs. In SSOs, the spectrum of disease from acute to subacute to chronic is characterized through clinical, biochemical, gross, and microscopic pathological findings [41]. Similar criteria (Table 4) may be used to differentiate acute and chronic toxicosis in CSLs [17,108,209]. Per-acute and acute-on-chronic descriptions are not available for any species.

## 9. Medical Treatment for DA Toxicosis

Treatment of individuals with acute DA toxicosis is focused on symptomatic and palliative care to manage seizures, maintain hydration and electrolyte balance, minimize patient discomfort, reduce inflammation, and prevent tissue damage. There are few therapies for toxin neutralization or elimination; thus, treatment is primarily supportive. The goal of treatment is to release recovered wildlife and discharge human ASP patients with a favorable prognosis by preventing long-term sequelae if possible. Wildlife with chronic toxicosis are not considered candidates for release. Except for CSLs and NFSs, treatment recommendations for marine wildlife with acute DA toxicosis are anecdotal or lacking. Successful treatment may be limited by DA’s potency, delayed presentation of individuals for care, and the challenge of antemortem diagnosis.

Seizure management in humans and pinnipeds with DA toxicosis involves a combination of anti-epileptic drugs (AEDs), typically phenobarbital and benzodiazepines [40,139,198]. Intoxicated marine mammals are usually anorexic; thus, parenteral treatment is commonly needed. The current recommendation for pinnipeds with acute DA toxicosis is a loading dose of phenobarbital (4 mg/kg BID IM for 2 d), followed by a maintenance dose (2 mg/kg BID IM or PO for 5 d) with the addition of midazolam, lorazepam, and/or diazepam (0.2–0.6 mg/kg PRN IM) for breakthrough seizures [209]. Lorazepam is more effective than diazepam for breakthrough seizures in CSLs [40], but a low dose of diazepam may assist with behavioral modification [257]. Anti-epileptic drug therapy is typically more successful in CSLs with acute toxicosis, as those with chronic disease often progress to a refractory state [17]. This was the case in a subadult CSL with suspected domoic acid-induced epileptic disease treated with phenobarbital (~1.5 mg/kg SID PO or IM) and lorazepam (0.1–0.2 mg/kg PRN IM) in managed care [257]. However, a Guadalupe fur seal with suspected chronic toxicosis was managed for two decades with phenobarbital alone (0.57–1.5 mg/kg BID PO or 0.57–0.8 mg/kg PRN IM) [137]. Dose adjustments are based on seasonal weight fluctuations, clinical status (i.e., abnormal behavior, reduced appetite, or breakthrough seizures), and trough phenobarbital levels [137,257]. Serum phenobarbital should be monitored at least annually, as well as when initiating or adjusting therapy or potential side effects are observed. A target serum level of 20–30 µg/mL is recommended since levels below 18 μg/mL often yield inadequate seizure control, while ataxia and sedation have been observed above 35 μg/mL [137,257]. Levetiracetam has also been used successfully for long-term epilepsy management in a few pinnipeds (C. Field, personal communication, December 2024). A CSL in managed care with progressive domoic acid-induced epileptic disease surgically transplanted with GABAergic interneuron porcine progenitor cells intrahippocampally demonstrated improved appetite, body weight, behavior, and seizure control [257,258]. Though xenotransplantation may be an effective therapy in pinnipeds with chronic DA toxicosis refractory to AEDs, the substantial expertise and resources required greatly limit its use, and xenotransplantation from domestic species may be inappropriate for wildlife intended for release. There are no AED dose recommendations for DA toxicosis treatment in other species, but phenobarbital and benzodiazepines may be appropriate [259,260]. Seizure control is critical for aquatic mammals that would be at risk of drowning; however, over-sedation can also occur, thus close monitoring and/or dry docking may be required for marine wildlife with unmanaged seizures and when medication is adjusted.

Individuals with DA toxicosis should receive palliative therapies. The purpose of fluid supplementation is to help maintain hydration and electrolyte balance, as well as support DA elimination from the body. Colloid fluids can be administered intravenously in hospitalized ASP patients and/or subcutaneously in rehabilitating or managing pinnipeds or fissipeds [40,137,198]. The ideal fluid dose is dependent on hydration and electrolyte status, but 20–25 mL/kg/day may be appropriate for anorexic pinnipeds and fissipeds [40,137,209]. Further correction of electrolyte imbalances, assisted feeding, and vitamin supplementation may be necessary [198,211]. Prokinetics (e.g., metoclopramide) can be administered to ASP patients known to have DA persisting in the GI tract to increase the speed of evacuation [198]. Endotracheal intubation may be required in patients with excessive bronchial secretions [197]. Antiemetics (e.g., maropitant) and antacids (e.g., famotidine) may be administered to treat GI upset [137]. Analgesics may be indicated for ASP patients with abdominal pain or headaches [198]. Induction of abortion is recommended in pregnant CSLs with acute DA toxicosis as the prognosis for the fetus is poor and the prognosis for the dam will improve [93,209]. Administration of dexamethasone sodium phosphate (40 mg or 0.25 mg/kg SID IM for 3 d) is usually an effective abortifacient in CSLs and should be followed by prostaglandin F2alpha (500 μg) or oxytocin if needed and possibly systemic antibiotics (e.g., ceftiofur crystalline free acid) to prevent pyometra.

Anti-inflammatory, antioxidant, and/or neuroprotective agents may aid in preventing or neutralizing DA-induced toxic impacts. The potential benefits of these agents have primarily been evaluated in model species and are as follows: (1) vitamin B6 (10 mg/kg) may reduce seizure activity [261], (2) troxuretin (150–225 mg/kg/d PO for 3 wk) may reduce memory deficits [262], (3) ursolic acid (100 mg/kg/d PO for 3 wk) may reverse memory deficits [263], (4) melatonin (10 mg/kg IP) may diminish neuronal damage, glial activation, and nitric oxide synthase induction [264], (5) purple sweet potato color (200 mg/kg/d PO for 4 wk) may attenuate cognitive deficits [265], (6) exogenous glutathione may inhibit apoptosis of cerebellar granular neurons [266], (7) ascorbate acid (10 mM for 24 h) may alleviate CNS deficits [267], (8) naringin (40–80 mg/kg IP) and sesamin extract (30 mg/kg SID PO for 3 d) may ameliorate seizures, cognitive dysfunction, CNS oxidative stress, and mortality rates [268,269], and (9) kynurenic acid (300–600 mg/kg IP after exposure) may reduce excitotoxic and convulsant stimulation [103,270,271,272,273]. Kynurenic acid, a generalized excitatory amino acid receptor antagonist, may also protect against DA-induced GI lesions. Neuroprotective strategies against DA may include (1) downregulation or knockdown of protein kinase C zeta (PKC-ζ) to reduce cognitive deficits and (2) upregulation or activation of CNS serotonergic 5-HT_1A_ receptors to attenuate seizures [274,275]. While these treatments were effective in models of DA toxicosis, they must be used with caution in marine wildlife and humans pending evaluation for safety and efficacy. The CNS antioxidant alpha-lipoic acid (ALA; 10 mg/kg SID SQ) and an anti-inflammatory dose of dexamethasone or prednisone (if no contraindications are present) are often administered to pinnipeds with acute toxicosis [209,276]. Though increased survival rates and decreased hippocampal damage were observed from a non-steroidal anti-inflammatory drug (NSAID) in models of acute DA toxicosis [194], NSAIDs may be unsafe in patients with renal compromise [277]. Moreover, three cases of acute renal failure after administration of an NSAID in CSLs undergoing rehabilitation for acute DA toxicosis implied a reduced renal capacity in these patients and underscored the importance of cautious NSAID use in DA toxicosis patients [277]. Treatment of acute DA toxicosis with exogenous glutamatergic antagonists is undergoing preclinical trials [198].

There are no published recommendations for the treatment of DA-induced cardiac disease or other severe extra-neural impacts (e.g., uterine torsion). Euthanasia should be considered when marine wildlife with DA toxicosis develop refractory seizures and/or substantial cardiomyopathy due to a poor prognosis for survival [209,211].

## 10. Prognosis

The prognosis related to DA exposure depends upon exposure dose, duration, history, underlying health status, time lapse since exposure, and stability of the population or species. Damage to GluR-containing organs may have enduring consequences on memory, navigation, social interactions, fecundity, cardiac output, and kidney function with cascading impacts on reproductive success, longevity, fitness, and survival [17,182].

There are no reports of stranded cetaceans with DA toxicosis surviving, or prognostic recommendations specific to fissipeds, seabirds, and sea turtles [211]. The individual prognosis from DA toxicosis has been best studied in CSLs, where major contributing factors include the type, severity, and timing of clinical signs, response to treatment, and underlying health status. Those with acute toxicosis who develop progressive seizures (i.e., domoic acid-induced epileptic disease) have a poorer prognosis than those with a gradual reduction in frequency [40]. Cognitive deficits manifest as anomalous diving and foraging patterns, which also negatively impact survivorship in CSLs with chronic toxicosis [233]. Scoring systems were developed and honed at The Marine Mammal Center (TMMC) in Sausalito, CA, USA to link clinical signs with prognosis. These “clinical assessment scores” or “NS” are performed following a week of AED treatment and cessation of AED treatment and aid clinicians with assessing patient progress, where higher scores are associated with a poorer outcome [40,209]. The best predictors of releasability are the third NS, change in NS over time, nutritional status on admittance, and if the patient begins eating during treatment [209]. In general, CSLs released after DA toxicosis treatment have a higher re-stranding rate (6% acute/chronic; 71% chronic) than those admitted for other reasons (0.5%), and more than 50% of CSLs with DA toxicosis are euthanized or die naturally [17,209]. Right dorsal hippocampal lesions correlate with poor navigational memory, and, thus, lesion presence and severity may be used as a prognostic marker [230]. There are no studies differentiating prognosis with long-term, low-level exposure vs. long-term sequelae to previously sublethal acute exposure. Since cognitive deficits were reversible after discontinuing long-term, low-dose exposure in model species [181], recovery may also be possible in humans and marine wildlife after suspending low-level DA ingestion. Marine wildlife deemed non-releasable are often euthanized due to a poor prognosis, but clinically stable, non-releasable pinnipeds may be transferred to public display or research facilities as ambassadors for their species and for long-term monitoring following environmental exposure [137,278]. The potential for gestational DA exposure must be considered in asymptomatic pinnipeds prior to placement in permanent care or release back into the wild since delayed but progressive neurobehavioral disease can occur [278]. The prognosis may be impacted by the increasing DA exposure dose, frequency, and duration expected with climate change and urbanization projections [71,72]. Threatened, endangered, and keystone populations may be the most vulnerable, particularly since DA often impacts prime-age adults whose reproductive success is important for population stability [186]. Prognosticating is limited by the challenge of diagnosis confirmation, as well as a lack of dose-effect and exposure history data.

## 11. California Sea Lions as Models of DA Toxicosis

**Figure 3:**
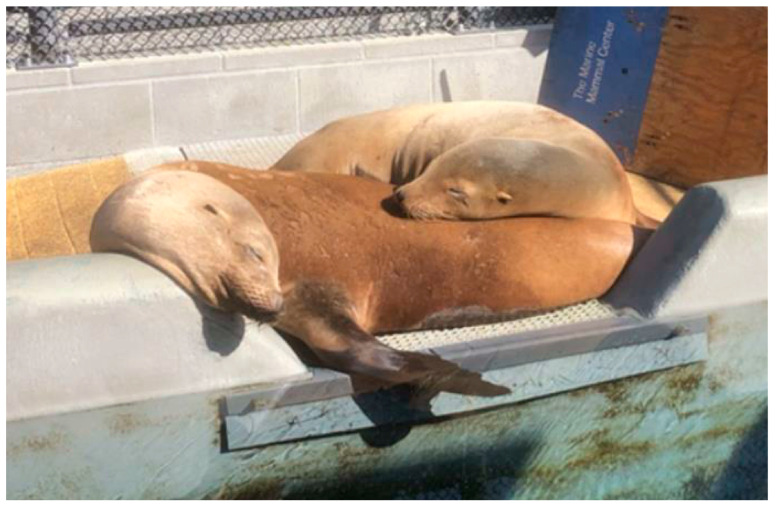
Two California sea lions (CSLs, *Zalophus californianus*) undergoing treatment for DA toxicosis at The Marine Mammal Center (TMMC, Sausalito, CA, USA), under NOAA permit #24359. This species is commonly rescued and transferred to a marine mammal rehabilitation center for DA-related care.

Extrapolation of findings from studies using laboratory models of DA toxicosis is often limited by the use of (1) taxa and exposure routes or doses that are not ecologically relevant, (2) healthy individuals that do not adequately represent sensitive subgroups, and (3) short study periods that do not characterize health impacts that may develop in long-lived species. California sea lions are effective mammalian sentinels of DA exposure due to their high trophic level status, susceptibility to toxin bioaccumulation and subsequent health effects, well-understood biology, capability of being researched under human-managed care, longevity, abundance, accessibility, similar DA sensitivity as humans, comparable physiology to humans, and unavoidable contact with well-documented seasonal blooms nearly annually [86,246,279,280,281,282]. California sea lions are commonly rescued and transferred to marine mammal rehabilitation facilities for DA-related care (Figure 3). Since CSLs move throughout marine environments, they can enhance stationary monitoring programs across spatiotemporal scales [283]. For example, CSLs may strand with acute DA toxicosis prior to increases of DA at collection sites (e.g., particulate DA from piers or shellfish), highlighting their utility as early warning tools [284]. However, DA-producing blooms do not always correspond to increased CSL stranding events [205]. For this reason, as well as the variability in DA uptake and depuration in commercially important seafood and the spatial and temporal heterogeneity in blooms and CSL movement patterns, such data is best used in tandem [205,284]. Finally, CSLs with DA toxicosis may serve as models of neurodegenerative and excitotoxic disorders also linked to overactivation of iGluRs in humans [285], including TLE, schizophrenia, autism spectrum disorder (ASD), Alzheimer’s disease, amyotrophic lateral sclerosis, MSG toxicosis, and β-Methylamino-L-alanine (BMAA) exposure [47,108,195,196,197,208,244,248,286,287,288,289,290,291].

## 12. Future Research

The primary focus of DA-related health research over the last three decades regarded the characterization and treatment of neurotoxicity in model species. Contemporary aims include better describing disease in extra-neural tissues, exposure risks in sensitive and high-risk groups (i.e., developing, aged, pregnant, or compromised individuals or those with long-term, low-level exposure), DA interactions with other biotoxins and contaminants, and the spectrum of disease along a temporal continuum and across the lifespan in humans and marine wildlife. Such studies can help provide an improved understanding of DA-induced clinical signs and survival, as well as inform diagnostic tests and treatments, thus improving the management of impacted individuals and conservation of affected populations.

## Figures and Tables

**Table 1:** Marine megafauna with suspected (S) or confirmed (C) environmental DA exposure, population-level impacts, or individual-level toxicosis due to exposure. Dashed line (-) represents no data available.

Species	Exposure	Population-Level	Individual-Level	Citation
Pinnipeds				
California sea lion (*Zalophus californianus*)	C	C	C	[40,93]
Stellar sea lion (*Eumetopias jubatus*)	C	-	S	[9,136]
Guadalupe fur seal (*Arctocephalus townsendi*)	C	S	C	[137,138]
South American sea lion (*Otaria byronia*)	C	-	-	[10]
Peruvian fur seal (*Arctocephalus australis*)	C	-	-	[10]
Northern fur seal (*Callorhinus ursinus*)	C	C	C	[9,139]
Harbor seal – Pacific, Scottish (*Phoca vitulina*)	C, C	-, S	C, S	[9,95,140,141,142]
Ringed seal (*Phoca hispida*)	C	-	-	[9,143]
Bearded seal (*Erignathus barbatus*)	C	-	-	[9,143]
Spotted seal (*Phoca largha*)	C	-	-	[9,143]
Ribbon seal (*Histriophoca fasciata*)	C	-	-	[9,143]
Pacific walrus (*Odobenus rosmarus*)	C	-	-	[9]
Fissipeds				
Sea otter— Southern, Northern (*Enhydra lutra*)	C, C	C, -	C, -	[9,41,42]
Cetaceans				
Bowhead whale (*Balaena mysticetus*)	C	-	-	[9,144]
Right whale- Northern, Southern (*Eubalaena glacialis, australis*)	C, C	S, S	-,-	[46,47,50]
Blue whale (*Balaenoptera musculus*)	C	-	-	[9,44]
Pygmy sperm whale (*Kogia breviceps*)	C	-	-	[45]
Dwarf sperm whale (*Kogia sima*)	C	-	-	[45]
Long-beaked common dolphin (*Delphinus capensis*)	C	-	C	[11,43]
Short-beaked common dolphin (*Delphinus delphis*)	C	-	C	[11,43]
Bottlenose dolphin (*Tursiops truncatus*)	C	-	C	[8,11,43,67,145,146,147]
Risso’s dolphin (*Grampus griseus*)	C	-	-	[11,43]
Harbor porpoise (*Phocoena phocoena*)	C	-	C	[9,11,95]
Dall’s porpoise (*Phocoenoides dalli*)	C	-	-	[11]
Minke whale (*Balaenoptera acutorostrata*)	C	-	C	[11,89]
Humpback whale (*Megaptera novaeangliae*)	C	-	-	[9,11,43,44]
Cuvier’s beaked whale (*Ziphius cavirostris*)	C	-	-	[11,43]
Gray whale (*Eschrichtius robustus*)	C	-	S	[11,43]
Fin whale (*Balaenoptera physalus*)	C	-	-	[11]
Northern right whale dolphin (*Lissodelphis borealis*)	C	-	-	[11]
Pacific white sided dolphin (*Lagenorhyncus obliquidens*)	C	-	-	[11]
Beluga whale (*Delphinapterus leucas*)	C	-	-	[9,148]
Long-finned pilot whale (*Globicephala macrocephalus*)	S	S	S	[49]
Seabirds				
Brandt’s cormorant (*Phalacrocorax penicillatus*)	C	S	C	[34,39]
Brown pelican (*Pelecanus occidentalis*)	C	S	C	[34,35,39]
Clark’s grebe (*Aechmophorus clarkii*)	C	-	C	[39]
Pacific loon (*Gavia pacifica*)	C	-	C	[39]
Red-throated loon (*Gavia stellata*)	C	-	C	[39]
Surf scoter (*Melanitta perspicillata*)	C	-	S	[39]
Common murre (*Uria aalge*)	C	-	C	[39,149]
White-winged scoter (*Melanitta deglandi*)	C	-	S	[39]
Double-crested cormorant (*Phalacrocorax auratus*)	C	-	C	[39]
Ring-billed gull (*Larus delawarensis*)	C	-	S	[39]
Cassin’s auklet (*Ptychoramphus aleuticus*)	C	-	C	[39,149]
Northern fulmar (*Fulmarus glacialis*)	C	-	S	[39,149]
Sooty shearwater (*Puffinus griseus*)	S	-	S	[150]
Marbled murrelet (*Brachyramphus marmoratus*)	C	S	C	[151]
**Marine Reptiles**				
Green sea turtle (*Chelonia mydas*)	C	-	S	[52,54]
Leatherback sea turtle (*Dermochelys coriacea*)	C	-	S	[53]

**Table 2:** Abnormal (↑ or ↓) routine blood work results reported after environmental DA exposure (BND = Bottlenose dolphin; CSL = California sea lion; GFS = Guadalupe fur seal; HS = Harbor seal; SB = Seabird; H = Human).

Blood Variable	Anomaly	Species	Citation
Complete Blood Count (CBC)			
Red Blood Cell (RBC)	↑	CSL	[218]
White Blood Cell (WBC)	↑	H, BND, CSL, GFS	[26,67,137,145,197,218]
Hemoglobin (HGB)	↑	CSL	[218]
Mean Corpuscular Volume (MCV)	↓	CSL	[218]
Platelet (PLT)	↑	CSL	[218]
Neutrophils	↑	CSL	[141,218]
Lymphocytes	↓	HS	[141]
Monocytes	↑	HS	[141]
Eosinophils	↑	CSL, BND	[67,145,209,217]
Hematocrit (HCT)	↑	CSL, GFS	[40,137,209]
Serum Biochemistry			
Serum Creatinine (sCr)	↑ or ↓	H, GFS, CSL	[26,137,197,218]
Blood Urea Nitrogen (BUN)	↑ or ↓	H, SB, CSL, GFS, CSL	[26,34,40,137,197,218]
Uric Acid (UA)	↑	SB	[34]
Creatine Kinase (CK)/ Creatine Phosphokinase (CPK)	↑	H, SB, CSL	[26,34,40,197]
Gamma-glutamyl transpeptidase (GGT)	↑	CSL	[218]
Alanine transaminase (ALT)	↑	CSL	[218]
Cholesterol (CHOL)	↓	CSL	[218]
Glucose (GLU)	↓	CSL	[218]
Total Bilirubin (TBili)	↑	CSL	[218]
Phosphorous (PHOS)	↓	CSL	[218]
Total Iron (TIRON)	↓	CSL	[218]
Calium (Ca^2+^)	↓	CSL	[218]
Sodium (Na^+^)	↓	CSL	[218]
Albumin (ALB)	↓	CSL	[218]
Total Protein (TP)	↑	GFS	[137]

**Table 3:** Summary of findings from studies utilizing molecular diagnostics after environmental DA exposure in California sea lions (CSL) and northern sea otters (NSO); CSF = cerebral spinal fluid; GluR = glutamate receptor.

Method	Species	Matrix	Findings	Citation
Gene microarray	CSL	Whole blood	Discriminated CSLs with DA toxicosis vs. other illnesses; Identified upregulation of inflammatory mediators and TNFAIP6 as a candidate biomarker of DA toxicosis	[248]
Peptidomics (MALDI-TOF with artificial neural networks)	CSL	Serum	This method can be a sensitive or specific diagnostic test for acute DA toxicosis	[252]
Proteomics (2D-GE/MS)	CSL	Plasma	Apolipoprotein E is a sensitive, but not specific biomarker of chronic DA toxicosis	[250]
Shotgun proteomics (LC-MS/MS)	CSL	CSF	Identified candidate biomarkers of DA toxicosis, as well as molecular mechanisms for neurodegeneration	[247]
Proteomics (MS)	CSL	CSF	Identified candidate biomarkers to diagnose and differentiate acute vs. chronic DA toxicosis	[256]
Polymerase Chain Reaction (PCR)	NSO	Whole blood	Evidence of persistent, low-level DA exposure to Kachemak Bay, Alaska sea otters; Noted differences in neurologic, cardiac, immune, and detoxification function gene expression in DA exposed vs. reference population	[222]
Immuno-histochemistry (IHC) + PCR	CSL	Heart	Pathway of DA-induced cardiac damage may involve direct activation of local GluRs and apoptosis	[100,104]
Immuno-fluorescence (IF)	CSL	Brain	Fluoro-jade staining did not identify ischemic neuronal degeneration per-acutely before standard HE staining	[101]
Immuno-fluorescence (IF)	CSL	Hippocampus	Correlated increased oxidative stress and glial activation with disease severity and glial activation and nitric oxide with the development of chronic toxicosis; Gliosis and alterations in glutamine synthetase may be part of mechanism for DA-induced seizures	[113]
Immuno-histochemistry (IHC)	CSL	Hippocampus	Oxidative stress is involved in acute and chronic DA toxicosis, whereas glutamine synthetase redistribution is only involved in chronic toxicosis	[117]
Immuno-cytochemistry (ICC)	CSL	Hippocampus	Supported similarity between human temporal lobe epilepsy (TLE) and chronic toxicosis	[244]

**Table 4:** Summary of criteria used to differentiate acute vs. chronic DA toxicosis in CSLs. (Condensed from [17,108,209]). BCS = body condition score.

	Acute DA Toxicosis	Chronic DA Toxicosis
**Brain** **histopathology**	None-hippocampal necrosis +/− involvement of other limbic system regions	Hippocampal atrophy +/− gliosis +/− involvement of other limbic system regions
**Clinical signs**	Ataxia, head weaving, seizures, tremors, coma, decreased responsiveness to stimuli, scratching behavior +/− good BCS	Intermittent seizures, episodic lethargy and inappetence, vomiting, central blindness, abnormal behaviors (stereotypic scratching, conspecific or human-directed aggression) +/− poor BCS
**Case history**	Strand in clusters (≥5 individuals within 48 h and 80 km) concurrent with a DA-producing bloom	Strand individually, possibly in an atypical location without a concurrent DA-producing bloom +/− previous treatment for acute toxicosis
**DA levels**	Not detectable-detectable	Not detectable

## Data Availability

No new data was created or analyzed in this study. Data sharing is not applicable to this article.
